# Increased Incidence of Hip Fractures During the COVID-19 Pandemic: A Retrospective Analysis From a Rural and Aged Community

**DOI:** 10.7759/cureus.87750

**Published:** 2025-07-11

**Authors:** Toshihiro Yamagata, Yasushi Terada, Katsutoshi Furukawa

**Affiliations:** 1 Division of Geriatric and Community Medicine, Faculty of Medicine, Tohoku Medical and Pharmaceutical University, Sendai, JPN; 2 Department of General Medicine, Cardiovascular Surgery, Shonai Amarume Hospital, Shonai, JPN

**Keywords:** aged populations, aging, covid 19, fall, fracture around hip, osteoporosis

## Abstract

The COVID-19 pandemic had a widespread impact on healthcare systems globally, particularly affecting older adults. This study was conducted to investigate long-term changes in hip fracture (HF) incidence before and during the COVID-19 pandemic in a rural and aged Japanese community. We retrospectively reviewed HF cases (femoral neck and trochanteric fractures) in patients aged ≥65 years at the Shonai Amarume Hospital in the Yamagata prefecture, Japan, from January 1, 2015, to December 31, 2024. Cases were divided into pre-pandemic (2015-2019) and pandemic (2020-2024) periods. Statistical analysis was performed using the Wilcoxon rank-sum and Kruskal-Wallis tests. A total of 78 HFs occurred in the pre-pandemic period and 134 during the pandemic. There were no significant differences in patient age or sex ratio between the two groups. Statistical analysis showed a significant increase in HF incidence during the pandemic (Wilcoxon p = 0.0461; Kruskal-Wallis p = 0.0495) in this rural and aged community. These findings suggest the need for targeted fall prevention and social support systems for older adults, particularly in aging regions, during public health crises.

## Introduction

Since the onset of the COVID-19 pandemic in early 2020, healthcare systems worldwide have faced significant strain. Older adults, particularly those in rural and aging communities, have been among the most affected [1]. COVID-19, caused by SARS-CoV-2, is now recognized as a multisystem disease with a broad range of complications, including musculoskeletal manifestations such as bone, joint, and muscle involvement [2,3]. Among musculoskeletal injuries, hip fractures (HFs) represent one of the most serious conditions, particularly in older populations due to their association with increased morbidity, mortality, and reduced functional independence [4].

Previous studies investigating changes in HF incidence during the pandemic have reported conflicting findings. For example, studies in the United Kingdom indicated a marked increase in HF cases (+61.7%) [4], whereas studies in Ecuador [5] and Japan [6] reported significant decreases. In contrast, a South Korean study reported no significant changes [7]. However, a limitation in most of these studies is their reliance on comparisons between only two single years (typically 2019 and 2020), which may not accurately reflect broader trends or long-term effects. Given this gap, we conducted a retrospective, multi-year analysis of HF incidence using data from 2015 to 2024 in a rural region in Japan with an aged demographic. Our aim was to evaluate whether the COVID-19 pandemic led to significant changes in HF incidence over time in this unique setting, and to contribute to evidence-based strategies for future healthcare planning in aging societies.

## Materials and methods

This retrospective study was conducted at the Shonai Amarume Hospital in Shonai Town in Yamagata prefecture, Japan, a rural and aged region. The study was approved by the Ethics Committee of Tohoku Medical and Pharmaceutical University (approval number: 2024-2-082-0000) and conformed to the provisions of the Declaration of Helsinki. The requirement for informed consent was waived due to the retrospective nature of this study. The total number of femoral neck and trochanter fractures for every year from 2015 to 2024 at Shonai Amarume Hospital was estimated as the total number of HF cases.

Eligibility criteria

Patients who were diagnosed with a femoral neck or trochanter fracture between January 1, 2015, and December 31, 2024, lived in Amarume town when they had the HF, and were aged 65 years or older when they had a HF, were included in the study. Patients aged 64 years or younger at the time of the HF were excluded.

Data analysis

The number of HF cases was compared between the pre-pandemic period (2015-2019) and the pandemic period (2020-2024) using the Wilcoxon rank-sum and Kruskal-Wallis tests. All statistical analyses were performed using SAS version 9.4 (SAS Institute Inc., Cary, North Carolina, United States).

## Results

A total of 212 HF cases were identified in individuals aged ≥65 years at Shonai Amarume Hospital between 2014 and 2024. Of these, 78 cases (21 men, 57 women) occurred during the pre-pandemic period (2015-2019) and 134 cases (36 men, 98 women) during the pandemic period (2020-2024). The mean patient age was 82.5±6.0 years in the pre-pandemic period and 82.9±4.3 years during the pandemic, with no statistically significant difference. Similarly, sex distribution did not significantly differ between the periods.

**Figure 1:**
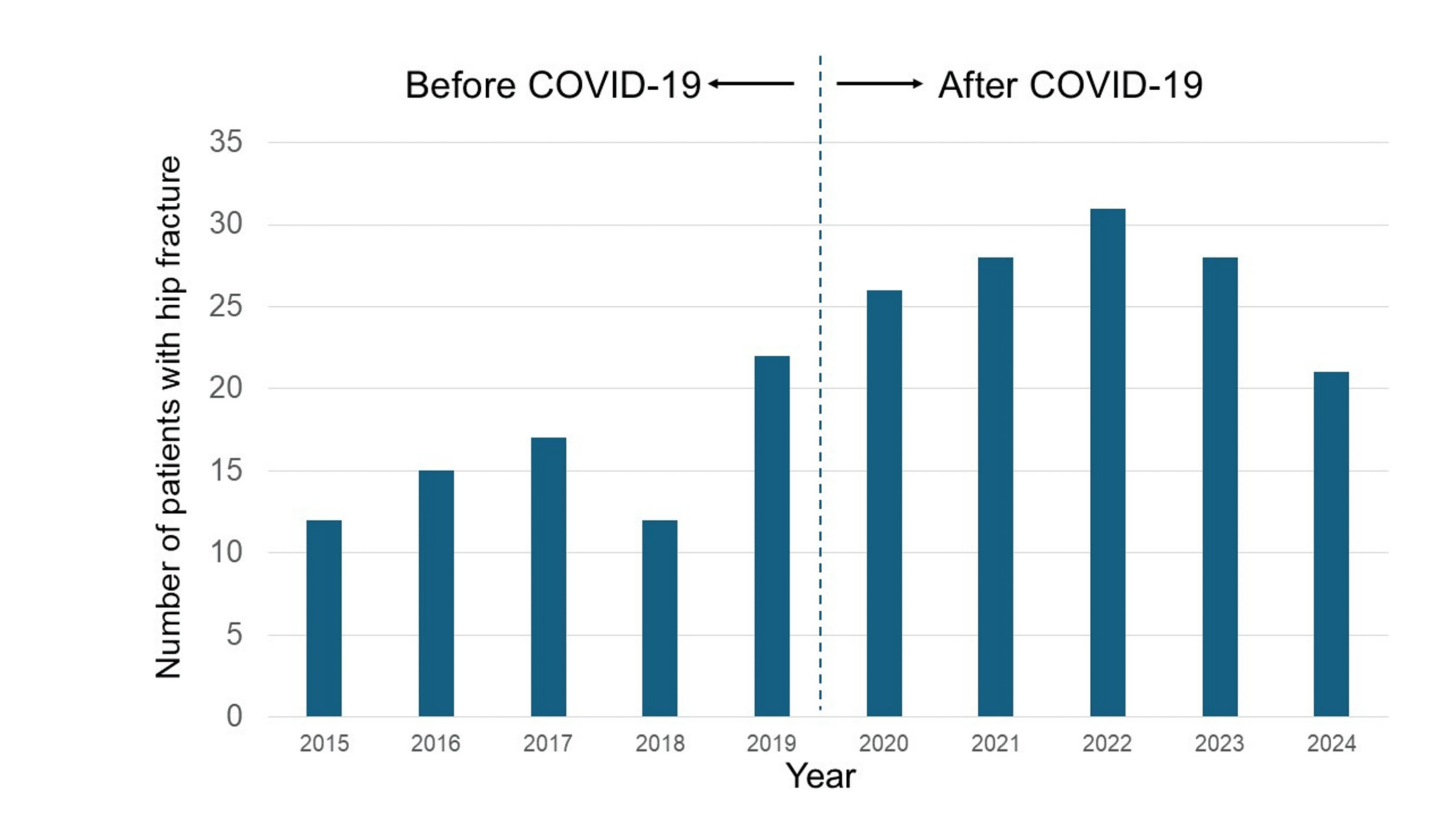
Annual number of patients with hip fractures from 2015 to 2024

Annual HF case numbers remained stable between 2015 and 2019 but increased markedly after 2020. Figure 1 illustrates the year-by-year trend, showing a consistent increase during the pandemic years.

Statistical comparison using the Wilcoxon rank-sum test revealed a one-sided p-value of 0.0461 (Z = -1.75), indicating a statistically significant increase in HF cases during the pandemic period. This was further supported by the Kruskal-Wallis test, which yielded a p-value of 0.0495 (T=5.84) (Table 1). Both results suggest a non-random and meaningful difference in HF incidence between the pre-pandemic and pandemic periods. These findings confirm a significant increase in HF incidence during the COVID-19 pandemic in this rural and aged community.

**Table 1:** Comparison of HF cases between pre-pandemic and pandemic periods HF: hip fracture

	Pre-pandemic period (2015-2019)	Pandemic period (2020-2024)	Wilcoxon rank-sum test		Kruskal-Wallis test	
			p-value	Z-value	p-value	T-value
Number of HF cases	78	134	0.0461	-1.75	0.0495	5.84
Annual mean	15.6	26.8				
Standard Deviation	4.16	3.7				

## Discussion

Our analysis revealed a significant increase in HF incidence among older adults during the COVID-19 pandemic in a rural and aged community. The COVID-19 pandemic brought unprecedented global challenges, leading to widespread lockdowns, disruptions in healthcare services, and significant shifts in population behavior. These changes prompted considerable interest in their potential effects on the epidemiology of various medical conditions, including fragility fractures like HFs.

While initial concerns existed regarding a potential increase in HF incidence due to factors like reduced physical activity, social isolation, and delayed medical care, the observed trends have been varied and complex. Studies conducted across different regions and healthcare systems have reported mixed findings regarding the change in HF occurrence during and after the pandemic. Some reports indicated a decrease in HF admissions, particularly during the initial strict lockdown periods. For instance, a study from New York noted a 29.9% decrease in HF cases during the early pandemic compared to the previous year, with patients also presenting later to the hospital [8]. Similarly, German data showed a consistent decline in most fracture types for individuals under 65, while HFs in those over 65 years of age remained largely unchanged [9]. A multi-center study in Japan observed a 6.8-7.5% reduction in HF patients in the early and late COVID-19 periods compared to the pre-COVID-19 period [5]. Conversely, other studies found no significant change or even slight increases in HF rates. A study in the United Kingdom reported no statistically significant difference in HF incidence during the first wave of the pandemic compared to the equivalent period in the previous year [10]. Similarly, findings from Niigata Prefecture, Japan, suggested a "minuscule" effect of the pandemic on the number and rate of fractures, with the proportion of indoor fractures increasing during emergency declarations [11]. Some studies also showed a drop in incidence that varied among hospitals and lockdown periods, indicating a non-uniform effect [12,13].

Shonai Town has one of the highest aging rates in Japan, with over 36% of its population aged ≥65 years, and its rate is extremely higher than the averages of Japan (29.3%) and the world (12%) [14-16]. The pandemic led to restrictions in mobility, reduced access to outpatient care and rehabilitation services, and limited support from caregivers. These factors likely increased fall risk and hindered recovery in this vulnerable population. Moreover, the psychological burden of social isolation and the physical deconditioning caused by prolonged inactivity during lockdowns may have contributed to the rise in HF [17]. In rural areas with limited medical resources, these challenges are often magnified. Our findings stress the urgent need for comprehensive fall-prevention strategies, especially during public health emergencies. Interventions such as home-based exercise programs, telehealth consultations, and community outreach could mitigate the effects of social isolation and inactivity. As Japan and other nations continue to experience rapid population aging, our study provides timely insight into how systemic disruptions like pandemics can disproportionately affect frail older populations in rural settings.

Limitations

Limitations of this study are as follows: (i) Single-center study: The data were collected from only one hospital (Shonai Amarume Hospital) in a specific rural and aged community in Japan. This limits the generalizability of the findings to other regions or populations with different demographics, healthcare systems, or pandemic responses. (ii) Retrospective design: As a retrospective analysis, the study relies on existing medical records, which may have limitations in data completeness or accuracy. It also cannot establish causality between the pandemic and the increase in hip fractures, only an association. (iii) Lack of detailed contributing factors: While the discussion suggests potential reasons for the increase (e.g., reduced mobility, social isolation, limited healthcare access), the study does not directly measure or quantify the impact of these specific factors on individual hip fracture cases.

## Conclusions

This study demonstrates a statistically significant increase in HF among older adults during the COVID-19 pandemic in a rural and aged Japanese community. By analyzing multi-year data, we provided more robust evidence than previous single-year comparisons. The increased incidence likely reflects pandemic-related lifestyle disruptions, including decreased mobility, limited healthcare access, and social isolation. These findings underscore the necessity for proactive public health measures, particularly in rural and aged communities, to support fall prevention and maintain musculoskeletal health during times of crisis. Policymakers must consider the unique vulnerabilities of older adults in remote areas when designing future health and disaster preparedness strategies.
